# Modification of early behavioural, physiological and neuropathological endpoints by syntaxin-6 knockout in a humanised P301S transgenic model of tauopathy

**DOI:** 10.1007/s00401-026-03009-2

**Published:** 2026-04-22

**Authors:** Elizabeth Hill, Jacqueline Linehan, Michael Farmer, Tatiana Jakubcova, Shyma Hamdan, Andrew Tomlinson, Silvia Purro, Fabio Argentina, Emma Jones, Nicholas Kaye, Craig Fitzhugh, Rohan de Silva, Sebastian Brandner, John Collinge, Thomas J. Cunningham, Simon Mead

**Affiliations:** 1 MRC Prion Unit at UCL, UCL Institute of Prion Diseases, Courtauld Building, 33 Cleveland Street, London, W1W 7FF UK; 2 School of Biochemistry, University of Bristol, Biomedical Sciences Building, Bristol, BS8 1TD UK; 3 Reta Lila Weston Institute, UCL Queen Square Institute of Neurology, London, WC1N 1PJ UK; 4 Department of Neurodegenerative Disease, UCL Queen Square Institute of Neurology, London, WC1N 3BG UK

**Keywords:** Tauopathies, Prion-like mechanisms, Neurodegeneration, Functional genetics

## Abstract

Genetically mediated increased expression of syntaxin-6, a SNARE protein involved in intracellular protein trafficking, is a proposed risk mechanism for progressive supranuclear palsy and sporadic prion disease. Increased syntaxin-6 protein levels are also causally associated with Alzheimer’s disease, suggesting it may have shared roles across multiple neurodegenerative diseases. However, no study has validated its functional role in tauopathies. To validate a role for syntaxin-6 in tauopathy pathogenesis, we knocked out syntaxin-6 in humanised P301S tauopathy mice. Mice underwent longitudinal rotarod testing, gait analysis, frailty and weight assessment, with neuropathological, biochemical and pathological analyses at 3 and 5 months. *Stx6*^+/+^;h*Tau*^P301S/P301S^ mice showed motor impairment from 1 month of age, which was partially rescued by syntaxin-6 knockout from months 1 to 4, with additional protection of gait at 5.5 months. Physiologically, syntaxin-6 knockout exerted a protective effect on weight trajectories and measures of frailty. Reduced neurodegeneration in the superficial cortex was observed at 3 months, as well as higher synaptic coverage at 5 months of age, supporting preserved neuropathological measures related to function. We further observed localised increases in tau pathology in the spinal cord and defined brain regions in young *Stx6*^*−/−*^*;*h*Tau*^P301S/P301S^ mice, despite total tau levels being comparable, in keeping with altered trafficking of pathological tau species with syntaxin-6 knockout. Despite a partial, early phenotypic rescue of functional measures, terminal endpoint comparisons were confounded by a 20% weight loss culling rule, as knockout mice maintained higher absolute weight. Taken together, this study functionally validates a role for syntaxin-6 in tauopathy pathogenesis, with syntaxin-6 knockout resulting in an early protective effect on multiple disease-relevant phenotypes in a humanised tauopathy model.

**Supplementary Information:**

The online version contains supplementary material available at 10.1007/s00401-026-03009-2.

## Background

Tauopathies comprise a class of neurodegenerative diseases, which are pathologically defined by tau inclusions in the brain [26]. Collectively, they are the result of the pathological corruption of tau, encoded by the *MAPT* gene, which has a primary, physiological role in binding and regulating microtubule assembly and stability, as well as axonal transport [8, 9, 47]. However, in the tauopathies, tau protein misfolds into distinct pathological assemblies, which seed further tau misfolding and spread intercellularly, whilst faithfully maintaining phenotypic properties [7, 15, 16, 30, 37]. Understanding modifiers of tau pathogenesis is essential for clarifying disease mechanisms, as there are likely shared molecular factors and pathways that influence susceptibility to, and progression or modification of, this group of neurodegenerative diseases.

Human genetics studies provide a powerful approach to identify genetic modifiers implicitly causal in human tauopathies and with higher translational potential [33–35]. Risk variants in and near to the *STX6* gene have been identified in multiple studies as genetic risk factors for progressive supranuclear palsy (PSP), the most common primary tauopathy [5, 6, 12–14, 20, 36]. An increase in syntaxin-6 expression has been proposed as the most likely genetic mechanism underlying disease risk [13, 14, 25]. Interestingly, there is also evidence that increased syntaxin-6 protein levels are causally associated with Alzheimer’s disease (AD) [48]. Furthermore, syntaxin-6 has also been implicated in disease risk in the most common human prion disease, sporadic Creutzfeldt–Jakob disease [18, 22, 23], suggesting it may have pleiotropic risk effects in neurodegenerative diseases. As there are fundamental similarities between prion disease and tauopathy pathogenesis, it is reasonable to hypothesise that there may be shared molecular modifiers of these processes.

Genetic data only provide suggestive evidence for the most likely gene driving disease risk at a locus. Recent work has functionally validated a role for syntaxin-6 in prion disease pathogenesis [19]. Specifically, mice with knockout of murine syntaxin-6 [24] showed reduced risk of developing disease after a low dose prion inoculation [19] suggesting syntaxin-6 influences early stages of prion disease in line with the human genetic studies [23]. Conversely, syntaxin-6 did not affect prion propagation kinetics or toxicity during established disease [19]. Furthermore, cellular studies implicated a role for syntaxin-6 in prion trafficking and export [19]. However, although these studies firmly establish syntaxin-6 as a modifier of prion pathogenesis, no study has functionally validated a role for syntaxin-6 in tauopathies. Given prion-like mechanisms are increasingly being recognised as being at play in the more common neurodegenerative diseases, it is likely syntaxin-6 may have a shared effect. Indeed, a role for syntaxin-6 in the secretion of tau monomer has been proposed [27]. However, no study has functionally validated a role for syntaxin-6 in tau pathogenesis in vivo*.*

The main aim of this work was to assess the functional effects of syntaxin-6 knockout in a humanised mouse model of tauopathy. To achieve this, we crossed *Stx6*^*−/−*^ mice with a widely used P301S 0N4R transgenic tauopathy mouse model [1] to assess alterations in disease progression as well as behavioural, physiological, neuropathological and biochemical outcome measures. This work supports syntaxin-6 being a modifier of tau pathogenesis in vivo*,* modulating predominantly early stages of the disease. Therefore, our results add to the growing body of evidence that syntaxin-6 acts as a pleiotropic prion/prion-like modifier across multiple neurodegenerative diseases, grounded in human genetics evidence.

## Materials and methods

### Ethics and research governance

Work with mice was performed under approval and license granted by the UK Home Office (Animals (Scientific Procedures) Act 1986), which conforms to UCL institutional guidelines and Animal Research: Reporting of In Vivo Experiments (ARRIVE) guidelines (www.nc3rs.org.uk/ARRIVE/). Full ethical review and research governance details are included in the Supplementary Methods.

### Animal studies

*Stx6*^+*/*+^ and *Stx6*^*−/−*^ mice were maintained on a C57BL/6N background with housing in pathogen-free, individually ventilated cages under standard environmental conditions (20–24 °C, 45–65% humidity, 12/12-h light/dark cycle). Irradiated feed and reverse osmosis water was supplied ad libitum. Genotyping for syntaxin-6 was performed using ear biopsies by polymerase chain reaction (PCR) as described in the Supplementary Methods. *Stx6*^*−/−*^ mice were crossed with Thy1-hTau.P301S mice [1] with copy count assays being used for genotyping, as detailed in the Supplementary Methods. The health of the mice was closely monitored throughout the study. Further details on the breeding strategy, study design, allocation to groups as well as censorship of animals are detailed in the Supplementary Methods.

### Neuropathological analysis

At 3 months, 5 months or upon reaching clinical endpoint, mice were euthanized by CO_2_ asphyxiation. Brains were dissected on the sagittal plane, with one hemisphere flash frozen for biochemical analyses and the other fixed in 10% (v/v) formal buffered saline for immunohistochemistry. Fixed tissue was processed into paraffin block and sectioned at 4 µm nominal thickness, prior to staining with AT8-biotin, MC1, synaptophysin, NeuN, GFAP or Iba1 targeting antibodies. The Gemini AS Automated Slide Stainer was used for haematoxylin staining using a conventional approach. Region-specific quantification of staining was performed using QuPath software (v0.4.3), with automated pixel classification to calculate the percentage area stained.

AT8 staining was also assessed in the spinal cord. Spinal columns were fixed in 10% (v/v) formal buffered saline and decalcified by rolling in 0.5 M EDTA solution for 7-10 days prior to dissection into segments and processing into paraffin blocks, with the thoracic region being used for AT8 staining.

Detailed protocols for sample preparation, staining and image analysis are provided in the Supplementary Methods.

### Biochemical assessment

 Brain homogenates (10% w/v) were prepared for western blot analysis to quantify total tau and pathological tau species. Samples were processed for western blotting with the AT8, PHF1 and K9JA antibodies being used for tau detection, as well as staining for β-actin as the loading control. Digital densitometry was conducted to quantify band intensities. Full details of the biochemical protocols including homogenate preparation, western blot procedures and antibodies are provided in the Supplementary Methods.

### Behavioural analysis in tauopathy mice

Rotarod and the ink blot test for gait assessment was performed in tauopathy mice with syntaxin-6 manipulation to assess for a modulatory effect on motor function. Frailty was assessed by observing the mice for the presence, absence and severity of the following 18 frailty characteristics: alopecia, loss of fur colour, dermatitis, righting reflex, coat condition, piloerection, eye discharge/swelling, microphthalmia, cataracts, nasal discharge, body condition, kyphosis, impaired gait during free walking, tremor, breathing rate/depth, tail stiffening, vestibular disturbance and distended abdomen. Full details can be found in the Supplementary Methods.

### Cell-based assays

A titration series of brain homogenates prepared from 2-month-old *Stx6*^+/+^;h*Tau*^P301S/P301S^ and *Stx6*^−/−^;h*Tau*^P301S/P301S^ mice was applied to tau biosensor cells. Aggregate count was measured using the Incucyte live cell imager. All cell culture procedures including transfection protocols and assay conditions are detailed in the Supplementary Methods.

### Statistical analysis

Sample sizes were determined based on power calculations for primary endpoints, with statistical analyses conducted using GraphPad Prism and InVivoStat. Where appropriate, data were transformed to meet parametric assumptions. Sex was included as a blocking factor with there being no consistent effect of sex on the phenotypes examined. Full statistical approaches, including criteria for exclusion and handling of missing data, are provided in the Supplementary Methods.

Full, detailed protocols for all procedures described here are available in the Supplementary Methods.

## Results

**Fig. 1:**
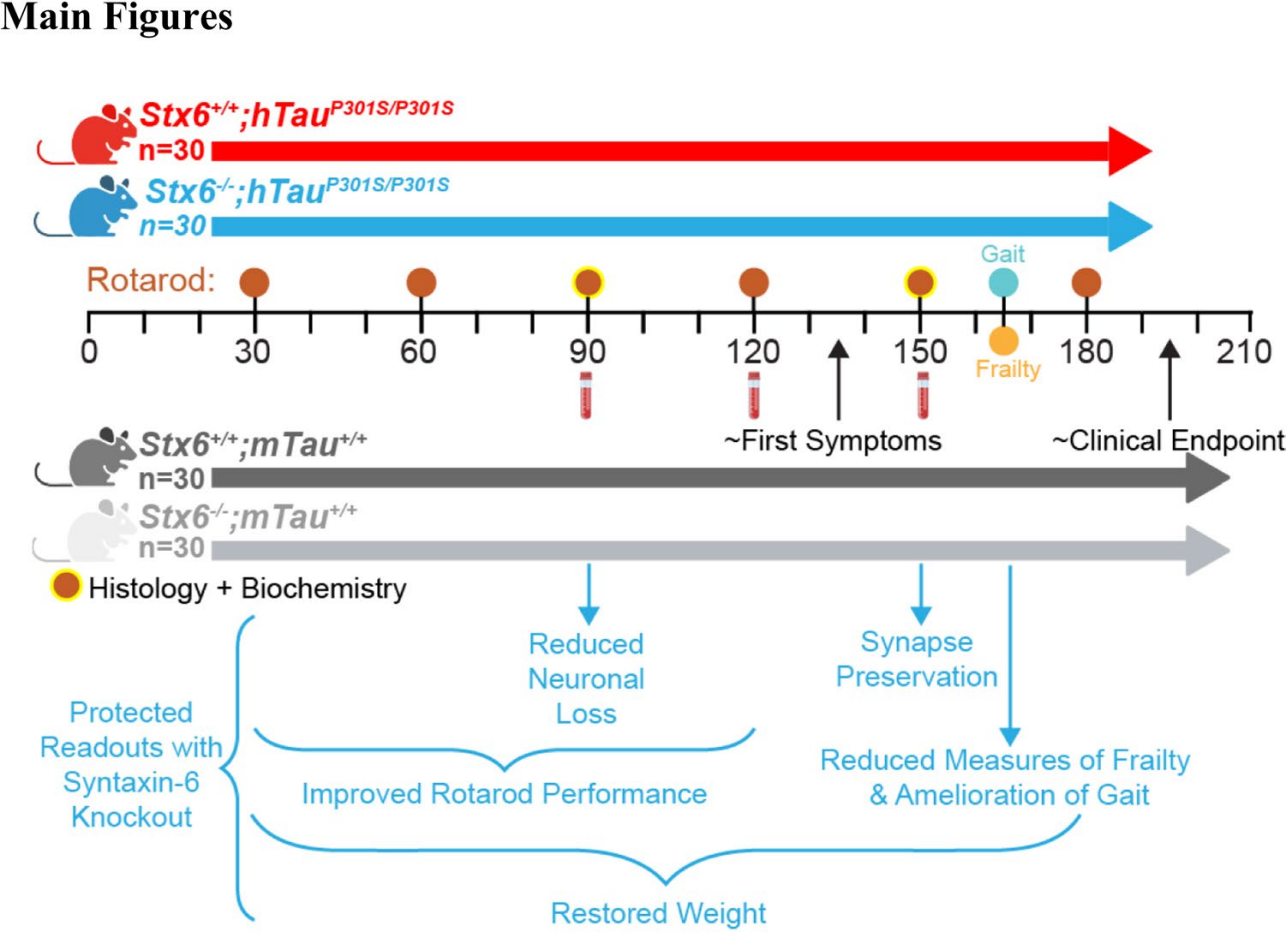
**Experimental design and summary of outcomes.** Overview of the experimental design summarising the four arms of the study and timeline of the different outcome measures assessed with a summary of the protective readouts with syntaxin-6 knockout in tauopathy mice below

Given the human genetics evidence, we hypothesised that syntaxin-6 may be a modifier of tauopathy pathogenesis in vivo. Therefore, to functionally validate and study a role for syntaxin-6 in tauopathy pathogenesis, we crossed *Stx6*^*−/−*^ mice with a transgenic P301S mutant tauopathy mouse model (h*Tau*^P301S/P301S^ mice) [1], which recapitulates many of the molecular and cellular features of human tauopathies. We subsequently conducted a longitudinal time course investigation of *Stx6*^+*/*+^*;*h*Tau*^P301S/P301S^ and *Stx6*^*−/−*^*;*h*Tau*^P301S/P301S^ mice, assessing numerous behavioural, clinical, neuropathological, biochemical and molecular phenotypes (Fig. 1).

### Protective effect of syntaxin-6 knockout on physiological and behavioural outcome measures in a transgenic P301S tauopathy model

**Fig. 2:**
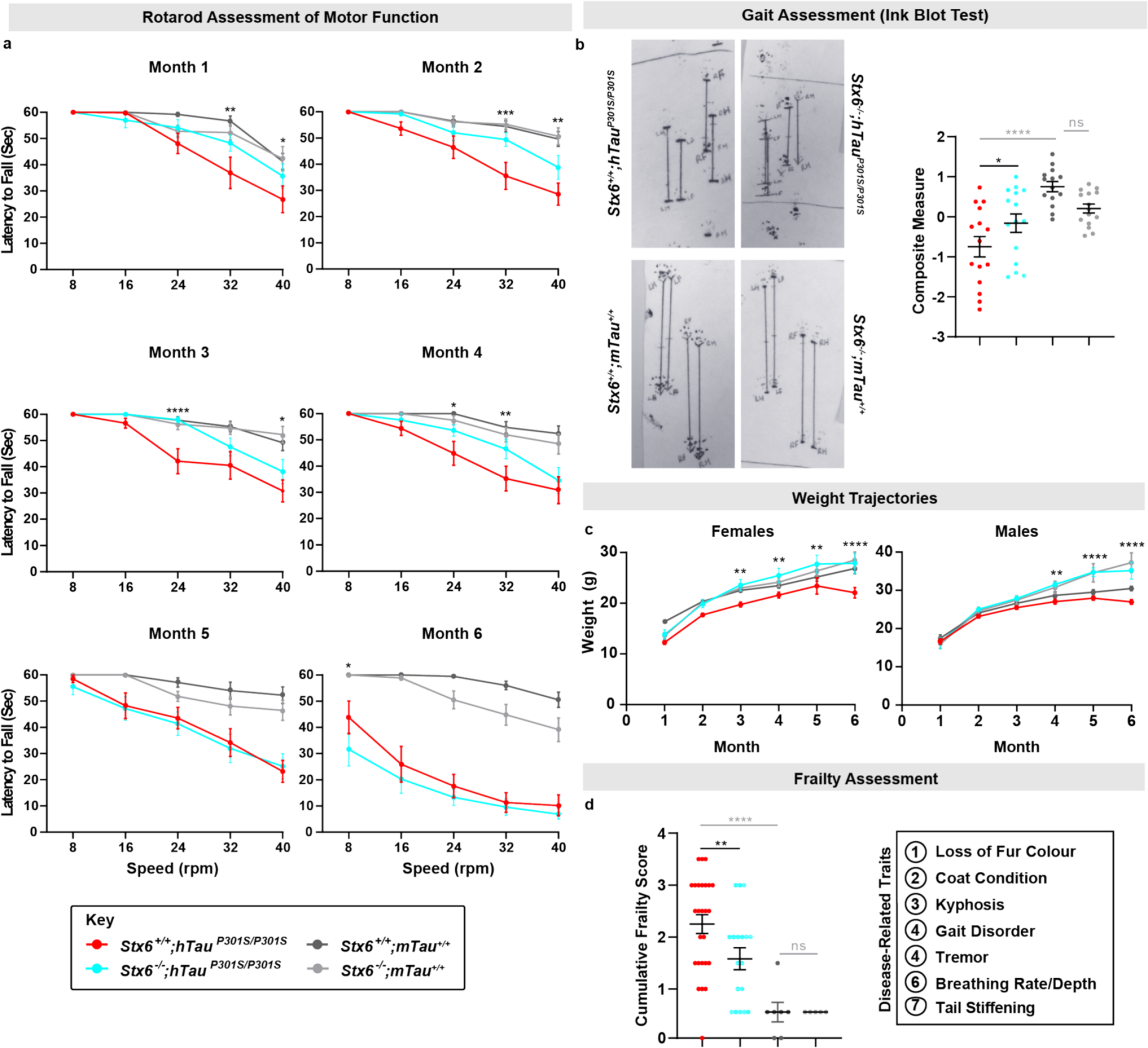
**Protective effect of syntaxin-6 knockout on behavioural and physiological outcome measures in tauopathy mice.** **a** *Stx6*^+/+^;h*Tau*^P301S/P301S^, *Stx6*^−/−^;h*Tau*^P301S/P301S^, *Stx6*^+/+^;m*Tau*^+/+^ and *Stx6*^−/−^;m*Tau*^+/+^ mice (*n* = 15/genotype, mixed sex) were subjected to 5 different speeds on a rotarod (8–40 rpm), receiving two trials/speed. The latency to fall (maximum trial length = 60 s) was recorded at each speed in the two trials. Data represent mean latency ± SEM. A 3-way repeated measures mixed-effect model was fitted to the data at each month with genotype and sex as factors and speed as a repeated factor. Stars relate to the results of the Fisher’s LSD post-hoc test of the preplanned comparison between *Stx6*^+/+^;h*Tau*^P301S/P301S^ and *Stx6*^−/−^;h*Tau*^P301S/P301S^ mice. **b** Gait assessment in 5.5-month-old mice (*n* = 15/genotype, mixed sex). Left: representative images of ink blots. Right: graph showing the mean composite measure ± SEM of disease-relevant gait parameters. A Z-score was calculated for each prioritised disease-relevant gait parameter per animal. Each dot represents the average Z-score/mouse, which were analysed using one-way ANOVA with genotype as the factor of interest and sex as a blocking factor followed by Fisher’s LSD test of preplanned comparisons. **c** Body weight of female (left) and male (right) mice at 1–6 months (*n* = 5–10/sex/genotype). Data are expressed as mean body weight (g) ± SEM. Statistical differences were determined using a 2-way repeated measures mixed model approach with genotype as the treatment factor and month as the repeated factor. As there was a significant interaction between genotype and month, each month was assessed separately using Fisher’s LSD test on the preplanned comparison between *Stx6*^+/+^;h*Tau*^P301S/P301S^ mice and *Stx6*^−/−^;h*Tau*^P301S/P301S^ mice. **d** Frailty assessment in *Stx6*^+/+^;h*Tau*^P301S/P301S^ (*n* = 26), *Stx6*^*−*/−^;h*Tau*^P301S/P301S^ (*n* = 18), *Stx6*^+/+^;m*Tau*^+/+^ (n = 7) and *Stx6*^−/−^;m*Tau*^+/+^ (*n* = 5) mice. Graph shows the cumulative frailty score [18 parameters each assessed using a 3-point score: normal (0), moderate (0.5) or severe (1)]. Each dot represents the cumulative frailty score for an individual mouse. Following rank transformation, the data were analysed using one-way ANOVA, with genotype as the treatment factor, and with sex and batch as blocking factors, followed by Fisher’s post-hoc LSD test of preplanned comparisons. *P < 0.05, **P < 0.01, ***P < 0.001, ****P < 0.0001

As h*Tau*^P301S/P301S^ mice exhibit a prominent, progressive motor impairment [39], we assessed whether syntaxin-6 knockout modifies rotarod performance (Fig. 2a). From month 1, *Stx6*^+/+^;h*Tau*^P301S/P301S^ mice displayed a motor defect compared to non-transgenic control *Stx6*^+/+^;m*Tau*^+/+^ mice, which persisted across the 6 months examined. In contrast, there was evidence for a partial rescue of the motor impairment in *Stx6*^*−/−*^*;*h*Tau*^P301S/P301S^ mice from months 1 to 4. Although rotarod performance converged in months 5 and 6, when mice were presenting with overt and debilitating clinical signs, syntaxin-6 knockout still exerted a protective effect in 5.5-month-old mice when we assessed gait as a less challenging assessment of motor dysfunction. Here, prioritised, disease-relevant gait parameters were harmonised into a composite score for each animal including fore distance, contralateral distance and ipsilateral apart distance (see methods). This validated a robust deficit in gait in *Stx6*^+/+^;h*Tau*^P301S/P301S^ mice relative to control *Stx6*^+/+^;m*Tau*^+/+^ mice (*P* < 0.0001) (Fig. 2b). In contrast, there was partial rescue of this impairment in *Stx6*^*−/−*^*;*h*Tau*^P301S/P301S^ mice (*P* = 0.033), providing an independent measure of functional amelioration with syntaxin-6 knockout.

As we had found that syntaxin-6 reduction mitigated some of the detrimental effects of tauopathy at the functional level, we also assessed physiological outcome measures. Weight trajectories provide an objective readout of general health and wellbeing, which were compromised in *Stx6*^+/+^;h*Tau*^P301S/P301S^ mice (Fig. 2c). Conversely, *Stx6*^*−*/−^;h*Tau*^P301S/P301S^ mice showed evidence of weight normalisation from month 3 in female mice, and from month 4 in male mice. Therefore, this serves as a positive correlate of disease amelioration. We also conducted an 18-point clinical exam in 5.5-month-old animals (derived from a validated frailty assessment [43]) (Fig. 2d). *Stx6*^+/+^;h*Tau*^P301S/P301S^ mice exhibited greater measures of frailty than the control *Stx6*^+/+^;m*Tau*^+/+^ mice (*P* < 0.0001), which was partially rescued in *Stx6*^−/−^;h*Tau*^P301S/P301S^ mice (*P* = 0.0052). In 6/7 disease-relevant frailty parameters, *Stx6*^+/+^;h*Tau*^P301S/P301S^ mice showed a higher proportion of animals with impairments (Supplementary Table 1).

Due to the severe phenotype of the h*Tau*^P301S/P301S^ mice, physical health checks were performed twice per week from 5 months of age. Upon noticing visible weight loss or abnormal gait (recorded as the time of first symptom), a weight baseline was established followed by weighing twice per week. Once an animal lost 15% of body weight, weighing was performed daily. Animals were culled when they reached the 20% weight loss endpoint or if they developed full hind limb paralysis prior to the weight loss endpoint. No differences were observed in these more subjective endpoint clinical outcome measures (Supplementary Table 2).

### Protective effect of syntaxin-6 knockout on neuropathological outcome measures related to function in a transgenic P301S tauopathy model

**Fig. 3:**
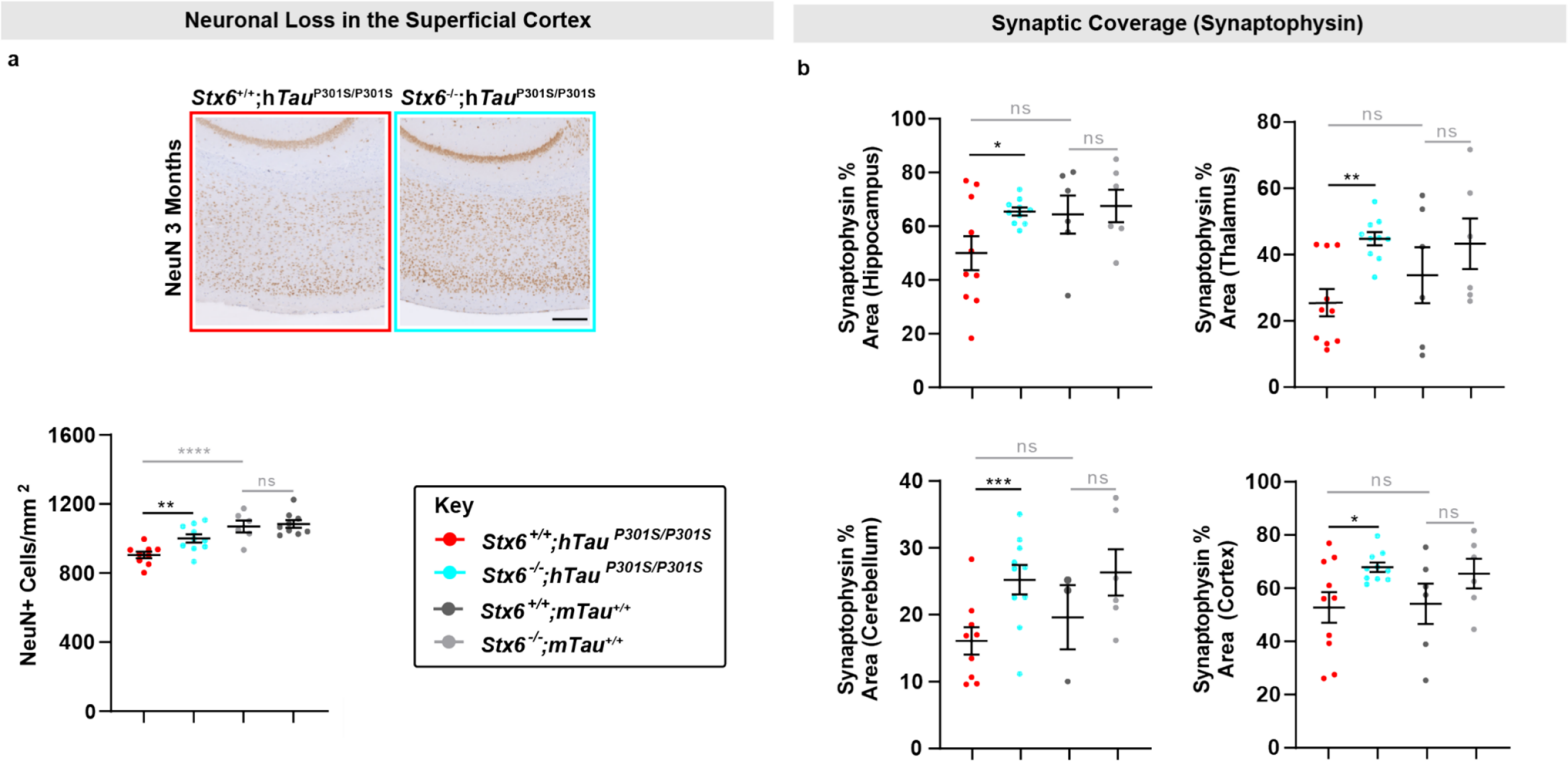
**Protective effect of syntaxin-6 knockout on neuropathological outcome measures related to function in tauopathy mice.** **a** Representative images of NeuN staining in the superficial cortex (500 μm deep from the cortical surface) in *Stx6*^+/+^;h*Tau*^P301S/P301S^ and *Stx6*^*−*/−^;h*Tau*^P301S/P301S^ mice at 3 months (top). Scale bar represents 250 μm. Quantification of image-based threshold analysis of NeuN staining in superficial cortex (below) (*n* = 6–10/genotype). One-way ANOVA was performed with genotype as the treatment factor and sex as the blocking factor, followed by Fisher’s LSD post-hoc test of preplanned comparisons. **b** Quantification of synaptophysin staining in different brain regions in *Stx6*^+/+^;h*Tau*^P301S/P301S^ and *Stx6*^*−*/−^;h*Tau*^P301S/P301S^ mice (*n* = 5–10/genotype, mixed sex) at 5 months. A 2-way repeated measures mixed model approach was used for statistical analysis using the unstructured covariance structure to model the within-subject correlations, with genotype as the treatment factor, brain region as the repeated factor and sex as a blocking factor. This was followed by planned comparisons on the predicted means to compare the effect of genotype on staining in the different brain regions. The brain stem was also assessed with there being no difference between *Stx6*^+/+^;h*Tau*^P301S/P301S^ and *Stx6*^*−*/−^;h*Tau*^P301S/P301S^ mice. *P < 0.05, **P < 0.01, ***P < 0.001, ****P < 0.0001

We hypothesised that we may observe a protective effect of syntaxin-6 knockout on neuropathological hallmarks related to function given the evidence of rescue at the behavioural level. Therefore, we quantified NeuN-positive cell density in the superficial cortex, which exhibits progressive neuronal loss in this tauopathy model [17]. We recapitulated this finding with 3-month-old *Stx6*^+*/*+^;*hTau*^P301S/P301S^ mice having a significant reduction in the number of NeuN-positive cells/mm^2^ relative to control *Stx6*^+*/*+^*;*m*Tau*^+*/*+^ mice (*P* < 0.0001), which was partially rescued with syntaxin-6 knockout (*P* = 0.0055) (Fig. 3a). Neuronal loss was comparable between *Stx6*^+/+^;h*Tau*^P301S/P301S^ and *Stx6*^*−*/−^;h*Tau*^P301S/P301S^ mice by 5 months (Supplementary Fig. 1a). Indeed, synaptic coverage has been reported in the literature to be reduced in the hippocampus, cortex, brain stem and cerebellum in this tauopathy mouse model [45]. We found higher synaptophysin staining coverage with syntaxin-6 knockout in the hippocampus (*P* = 0.0151), thalamus (*P* = 0.0023), cerebellum (*P* = 0.0007) and the cortex (*P* = 0.0127) at 5 months compared to *Stx6*^+/+^;h*Tau*^P301S/P301S^ mice (Fig. 3b). However, neuroinflammatory phenotypes did not correlate with the protective effect of syntaxin-6 knockout (Supplementary Fig. 1b-c). Therefore, these results suggest a partial rescue of neurodegeneration-related neuropathological hallmarks.

### The effect of syntaxin-6 knockout on pathological tau species

**Fig. 4:**
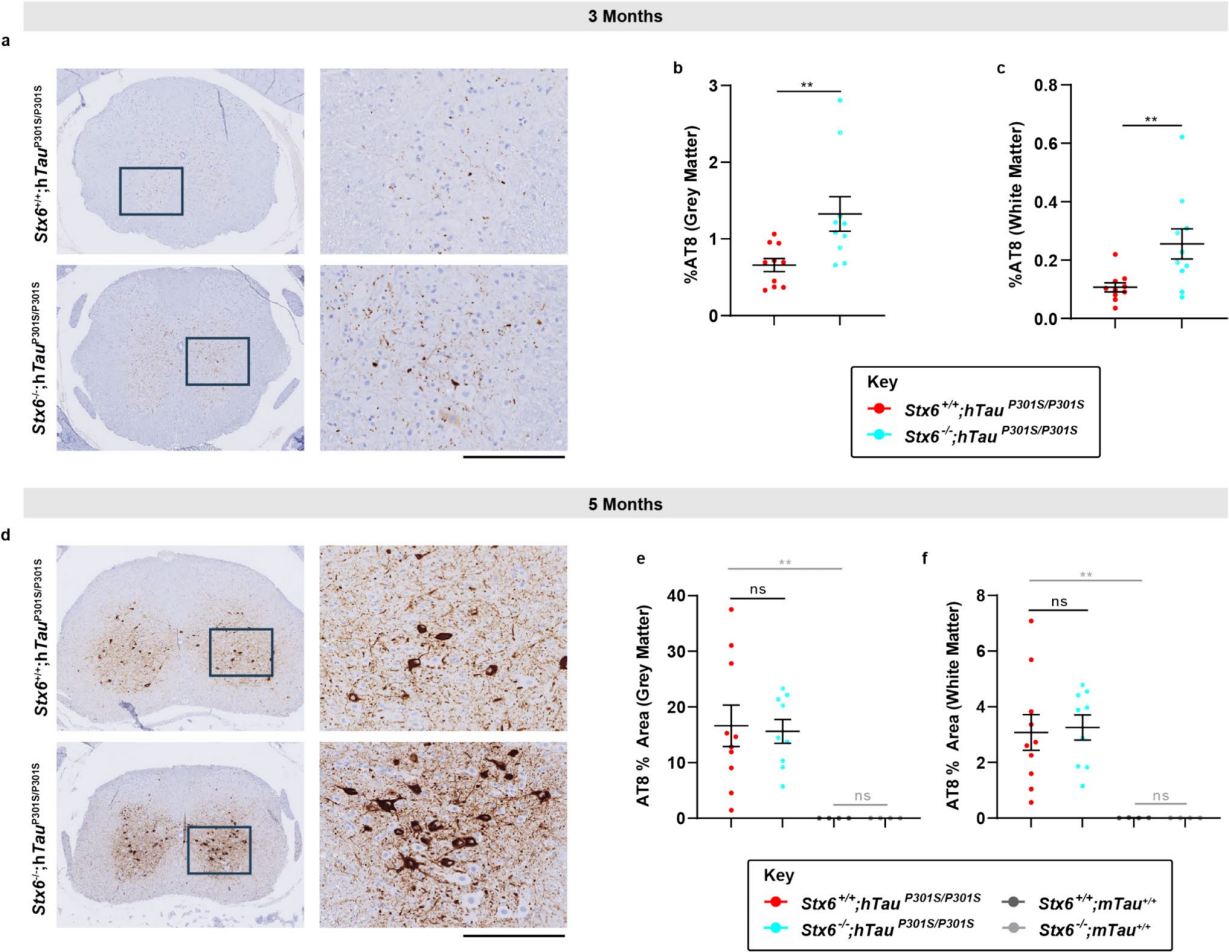
**Increased AT8-positive tau in the spinal cord of ***Stx6*^−/−^;h*Tau*^P301S/P301S^ **mice at early disease stages**. Spinal cords at the thoracic level of tauopathy mice were stained with an antibody detecting the pSer202/pThr205 phospho-epitope of tau (AT8) at 3 and 5 months of age. **a**, **d** Representative images of staining in *Stx6*^+/+^;h*Tau*^P301S/P301S^ and *Stx6*^−/−^;h*Tau*^P301S/P301S^ mice at 3 months (**a**) and 5 months (**d**). Scale bar, 800 µm (overview), 200 µm (zoom). **b**, **c**, **e**, **f** Corresponding quantification of AT8 staining density in the grey matter (b, e, H-shape or butterfly pattern) and the white matter (c, f, outer ring) (*n* = 10/ main experimental arm groups). A 2-way repeated measures mixed model approach was used for statistical analysis using the unstructured covariance structure to model the within-subject correlations, with genotype as the treatment factor, brain region as the repeated factor and sex as a blocking factor. This was followed by planned comparisons on the predicted means to compare the effect of genotype on staining in the different regions. This analysis was performed post-rank transformation for the 3-month data. Two animals were excluded due to anatomical deviations. Images are representative, selected to demonstrate neuronal morphology and staining quality, including the high signal-to-noise ratio achieved. Data represent means ± SEM. **P < 0.01

**Fig. 5:**
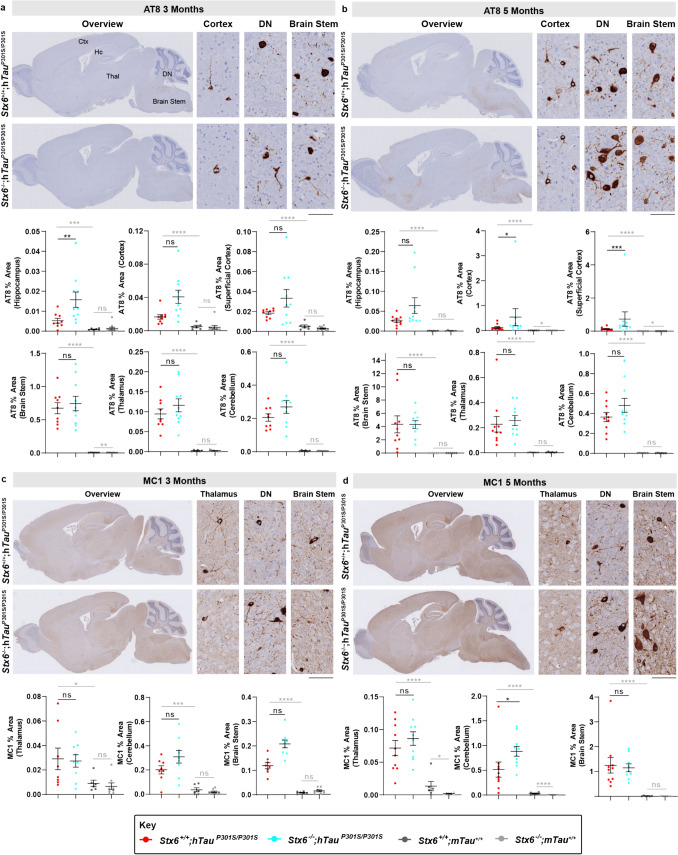
**Immunohistochemical assessment of pathological tau reveals distribution differences in ***Stx6*^−/−^;h*Tau*^P301S/P301S^ **mice relative to ***Stx6*^+/+^;h*Tau*^P301S/P301S^ **mice**. **a**, **b** Brain sections of tauopathy mice were stained with an antibody detecting the pSer202/pThr205 phospho-epitope of tau (AT8) at 3 months of age (**a**) or 5 months of age (**b**). Whole brain sections (left) as well as representative images from the cortex, the dentate nucleus (DN) of the cerebellum and brain stem are shown. Quantification of AT8 staining density is indicated below (*n* = 10/main experimental arm groups). Following rank transformation, a 2-way repeated measures mixed model approach was used for statistical analysis using the unstructured covariance structure to model the within-subject correlations, with genotype as the treatment factor, brain region as the repeated factor and sex as a blocking factor. This was followed by planned comparisons on the predicted means to compare the effect of genotype on staining in the different brain regions. All brain regions assessed are shown as all had a disease-associated increase in staining relative to *Stx6*^+/+^;m*Tau*^+/+^mice. **c**, **d** Brain sections of tauopathy mice were stained with a tau conformational antibody (MC1) at 3 months of age (**c**) or 5 months of age (**d**). Whole brain sections (left) as well as representative images from the thalamus, the DN and the brain stem are shown. Quantification of MC1 staining density is indicated below (*n* = 10/main experimental arm groups). Following rank transformation, a 2-way repeated measures mixed model approach was used for statistical analysis using the unstructured covariance structure to model the within-subject correlations, with genotype as the treatment factor, brain region as the repeated factor and sex as a blocking factor. This was followed by planned comparisons on the predicted means to compare the effect of genotype on staining in the different brain regions. The three brain regions with a disease-associated increase in staining at 3 months are shown. At 5 months, there was also a disease-associated increase in staining in the cortex and hippocampus but there were no differences between *Stx6*^+/+^;h*Tau*^P301S/P301S^ and *Stx6*^−/−^;h*Tau*^P301S/P301S^ mice. Images are representative, selected to demonstrate neuronal morphology and staining quality, including the high signal-to-noise ratio achieved. Data represent means ± SEM. Scale bar, 2 mm (overview), 100 µm (high magnification). *P < 0.05, **P < 0.01, ***P < 0.001, ****P < 0.0001

**Fig. 6:**
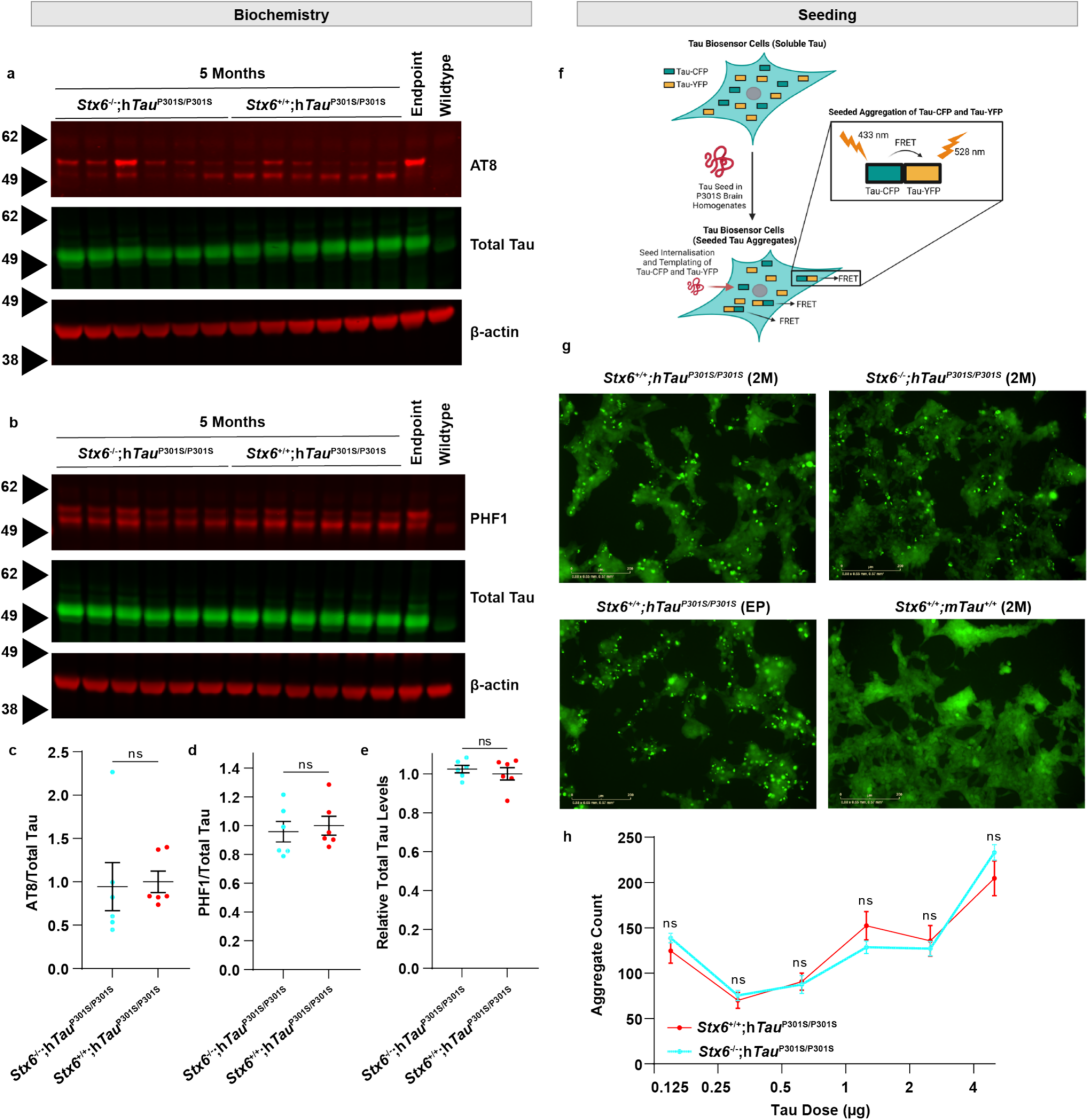
**No differences in the biochemical and cell-based assessment of total pathological tau species.** **a** Immunoblot of total brain homogenates of 5-month-old animals using an antibody detecting the pSer202/pThr205 phospho-epitope of tau (AT8, top), an antibody against total tau (K9JA, middle) and β-actin as a loading control (bottom). **b** Immunoblot of brain homogenates of 5-month-old animals using an antibody detecting Ser396/404 (PHF1), an antibody against total tau (K9JA, middle) and β-actin loading control (bottom). Brightness and contrast were optimally adjusted. **c** Quantification of the AT8 signal intensity normalised to the β-Actin loading control corrected by the total tau band intensity. This was finally normalised to the average intensity values of the *Stx6*^+*/*+^*;*h*Tau*^P301S/P301S^ mice. Line and error bars: mean ± SEM with each dot representing an individual mouse. Significance levels based on results of an unpaired *t*-test (*n* = 6/genotype). **d** Quantification of PHF1 intensity with normalisation and statistics as described above (*n* = 6/genotype). **e** Quantification of total tau (*n* = 6 animals/genotype), with each dot representing an average of 3 technical replicates across gels. No differences were also found at 3 months or endpoint (data not shown). **f**–**h** A titration series of brain homogenates prepared from 2-month-old *Stx6*^+/+^;h*Tau*^P301S/P301S^ and *Stx6*^−/−^;h*Tau*^P301S/P301S^ mice was applied to tau biosensor cells. Graph shows aggregate count 48 hrs post-treatment using the Incucyte live cell imager. Statistics refer to the results of two-way ANOVA with tau dose and genotype as factors, followed by Fisher’s LSD test. There was a significant effect of tau dose (*P* < 0.0001) but no effect of genotype. *P < 0.05, **P < 0.01, ***P < 0.001, ****P < 0.0001

To explore whether differences in pathological tau species were associated with the prominent functional rescue with syntaxin-6 knockout, we assessed established pathological tau epitopes by either immunohistochemistry or biochemistry. Surprisingly, immunohistochemical characterisation of AT8-positive phospho-tau in the spinal cord was robustly increased in *Stx6*^*−*/−^;h*Tau*^P301S/P301S^ mice relative to *Stx6*^+/+^;h*Tau*^P301S/P301S^ mice at 3 months but not at 5 months (Fig. 4). There was also a subset of brain regions with elevated AT8-positive phospho-tau or MC1-positive misfolded tau in *Stx6*^−/−^;h*Tau*^P301S/P301S^ mice (Fig. 5). However, we did not observe any differences in AT8-positive or PHF1-positive phospho-tau species in total brain homogenates by western blot at either 3 months or 5 months (Fig. 6a–d) and total tau levels were comparable (Fig. 6a, b, e). There were also no differences in seeded tau aggregation when we applied brain homogenates prepared from 2-month-old mice to HEK tau biosensor cells [21] (Fig. 6f–h). Given that we observed localised increases of tau pathology in *Stx6*^−/−^;h*Tau*^P301S/P301S^ mice by immunohistochemistry despite the total biochemical load of phospho-tau species and seeding-competent species remaining constant, these results are in keeping with altered trafficking of pathological tau species with syntaxin-6 knockout, with a higher proportion of tau species being in a highly aggregated state. Furthermore, these results support an uncoupling of the functional outcome measures with the levels of pathological tau, which has been observed previously (see discussion).

Collectively, our results have demonstrated evidence for a protective effect of syntaxin-6 knockout on numerous behavioural, physiological and neuropathological outcome measures, as well as changes in the distribution/aggregation state of pathological tau species in keeping with an intracellular trafficking mechanism of action.

## Discussion

We provide evidence that syntaxin-6 is a modifier of tau pathogenesis in vivo, providing functional support for previous GWAS findings [5, 6, 12–14, 20, 36]. Specifically, we show that syntaxin-6 knockout in a transgenic tauopathy mouse model resulted in an amelioration of numerous physiological, behavioural and neuropathological outcome measures. Considering syntaxin-6 has already been implicated in prion disease pathogenesis [19], this study thus increases the relevance of syntaxin-6 to multiple neurodegenerative diseases which share prion-like features.

The protective effect of syntaxin-6 knockout in a humanised tauopathy model aligns with previous work in the prion field where knockout of syntaxin-6 in experimental mice reduced the risk of disease transmission [19]. Despite being different experimental models (genetic mouse model vs. acquired, inoculation mouse model), the direction of the effect consistently converges on protection with reduced syntaxin-6 expression. Future work may involve exploring the role of syntaxin-6 in a mouse model inoculated with tau seeds to allow more precise parallels to be drawn between the syntaxin-6 in vivo functional genetics studies in prion and tau contexts. Our work additionally aligns with the human genetic data linking increased expression of syntaxin-6 to higher disease risk in tauopathies [13].

Despite the mitigation of motor deficits, frailty measures and neurodegeneration-related pathology with syntaxin-6 knockout, interestingly, we observed increased localised AT8- and MC1-positive tau pathology, whilst total phospho-tau and seeding-competent tau levels remained unchanged. This suggests an altered pathological tau distribution akin to previous reports showing that prion aggregates adopt altered morphology and distribution in cellular models with perturbed syntaxin-6 expression [19]. Given that syntaxin-6 has been implicated in prion export [19], these neuropathological findings can be consolidated with reduced tau export with syntaxin-6 knockout. This aligns with prior reported cellular evidence that syntaxin-6 and syntaxin-8 are involved in the export of tau monomer [27]. Furthermore, the observed dissociation between tau burden and toxicity aligns with previous studies showing functional preservation despite persistent pathology [2, 3, 10, 38, 42, 44, 46].

Interpretation of the clinical endpoint requires caution because the protocol defined euthanasia at 20% weight loss and the *Stx6*^−/−^;h*Tau*^P301S/P301S^ mice maintained higher body weight throughout the late disease phase. As a result, these animals reached the endpoint at a higher absolute weight and may have survived longer under alternative endpoint definitions. This provides a plausible explanation for the lack of genotype differences at the terminal clinical assessment despite multiple earlier benefits of syntaxin-6 knockout. Indeed, the prominent functional rescue observed in the more sensitive functional tests demonstrates that syntaxin-6 knockout favourably modifies the disease course in this transgenic tauopathy model.

Targets supported by human genetics evidence may offer higher translational value [29, 33–35]. Although tau lowering offers a genetically validated therapeutic approach in tauopathies with preclinical efficacy [11, 31, 32], extending our therapeutic repertoire would be valuable. Our results raise questions about whether syntaxin-6 could offer a target for drug development in tauopathies. However, given the modesty of the effects, and the lack of modification of the clinical endpoint, we suggest that more work needs to be done before a compelling argument can be made. Here, it is important to note that we did utilise a very severe mouse model of tauopathy with rapid clinical progression. This may have hindered our ability to observe protective effects of higher magnitude.

Limitations of this work include the use of genetic mouse model of tauopathy overexpressing a rare FTD-associated mutation [4, 28, 41, 49], resulting in a disconnect with the risk-conferring effect identified in sporadic human aetiology. Of note, this model generates tau filaments which are structurally distinct to known wild-type tau filaments in human tauopathy patients (although tau filament structures in FTD patients harbouring the P301S mutation are not yet known) [40]. Nevertheless, due to the lack of “better” models, this work provides an important proof-of-concept to inform future studies. It should also be acknowledged that the transgenic mouse model used in this study expresses murine *Stx6* in the context of human P301S tau. Accordingly, the extent to which these findings reflect human STX6-tau interactions in humans remains to be determined.

Taken together, we propose syntaxin-6 as a modifier of tauopathy pathogenesis in vivo, advancing our overall understanding of the relevance of syntaxin-6 to neurodegenerative diseases as a pleiotropic risk factor.

## Supplementary Information

Below is the link to the electronic supplementary material.

Supplementary material (DOCX 2806 KB)

Supplementary material (DOCX 62 KB)

## Data Availability

Lead contact: Requests for further information and resources should be directed to and will be fulfilled by the lead contact, Simon Mead (s.mead@prion.ucl.ac.uk).

## Materials availability

This study did not generate new unique reagents.

## Code availability

This paper does not report original code. Any additional information required to reanalyse the data reported in this paper is available from the lead contact upon request.
