# Supplementary material for: Modification of early behavioural, physiological and neuropathological endpoints by syntaxin-6 knockout in a humanised P301S transgenic model of tauopathy
Source: Acta Neuropathol. 2026 Apr 22;151(1):44. doi: 10.1007/s00401-026-03009-2 (PMC13102793; doi:10.1007/s00401-026-03009-2)
Supplement: Supplementary file 1 — Supplementary material (DOCX 2806 KB) [file 401_2026_3009_MOESM1_ESM.docx]

# Supplementary Figures


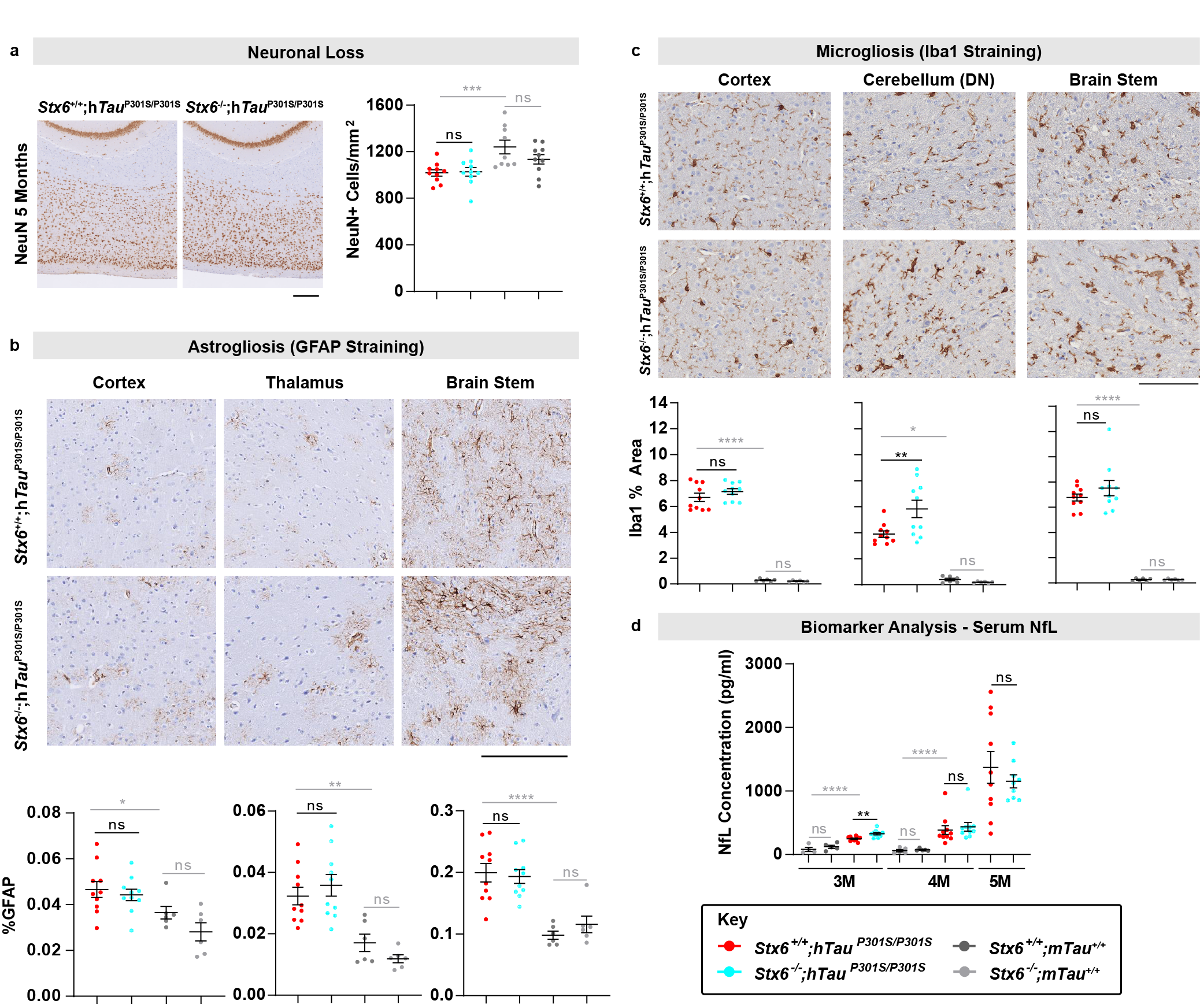


### Supplementary Fig. 1 No Differences in Neuronal Loss or Neuroinflammation at 5 months nor Serum NfL. (a) Representative images of NeuN staining in the superficial cortex (500 μm deep from the cortical surface) in *Stx6*^+/+^;h*Tau*^P301S/P301S^ and *Stx6^-^*^/-^;h*Tau*^P301S/P301S^ mice (n=10/genotype for main experimental comparison, mixed sex) at 5 months (left). Scale bar represents 250 μm. Quantification of image-based threshold analysis of NeuN staining in the superficial cortex (right). One-way ANOVA was performed with genotype as the treatment factor and sex as a blocking factor, followed by Fisher’s LSD post-hoc test of pre-planned comparisons. (b) Astrogliosis was assessed by staining brain sections from 5-month-old mice with an anti-GFAP antibody. Quantification of the staining is shown for brain regions with a disease-associated increase in staining (cortex, thalamus and brain stem) (n=10/genotype). Following rank transformation, a 2-way repeated measures mixed model approach was used for statistical analysis using the unstructured covariance structure to model the within-subject correlations, with genotype as the treatment factor, brain region as the repeated factor and sex as a blocking factor. This was followed by planned comparisons on the predicted means to compare the effect of genotype on staining in the different brain regions. (c) Microgliosis was assessed by staining brain sections from 5-month-old mice with an anti-Iba1 antibody. Quantification of staining from the cortex, the dentate nucleus (DN) region of the cerebellum and brain stem are shown (n=10/genotype). Following rank transformation, a 2-way repeated measures mixed model approach was used for statistical analysis using the unstructured covariance structure to model the within-subject correlations, with genotype as the treatment factor, brain region as the repeated factor and sex as a blocking factor. This was followed by planned comparisons on the predicted means to compare the effect of genotype on staining in the different brain regions. Data in graphs represent means ± SEM. The hippocampus and thalamus also showed a disease-associated increase in staining in tauopathy mice, with there being no effect of syntaxin-6 knockout (data not shown). (d) Neurofilament light chain (NfL) assessment in serum at 3 months, 4 months and 5 months. *P < 0.05, **P < 0.01, ***P < 0.001, ****P < 0.0001.

# Supplementary Tables

### Supplementary Table 1. Percentage Distribution of Frailty Scores in *Stx6*^+/+^;h*Tau*^P301S/P301S^ and *Stx6*^-/-^;h*Tau*^P301S/P301S^ Mice. Table showing disease-related frailty parameters and the percentage of animals of each genotype that were recorded to have no deficit (score = 0), a mild deficit (score = 0.5) or a severe deficit (score = 1).

|  | ***Stx6*^+/+^;h*Tau*^P301S/P301S^ Mice** | | | ***Stx6*^-/-^;h*Tau*^P301S/P301S^ Mice** | | |
| --- | --- | --- | --- | --- | --- | --- |
| **Parameter** | **Normal** | **Moderate** | **Severe** | **Normal** | **Moderate** | **Severe** |
| Fur colour loss | 34.6 | 65.4 | 0 | 50 | 50 | 0 |
| Coat condition | 65.4 | 34.6 | 0 | 72.2 | 27.8 | 0 |
| Kyphosis | 26.9 | 69.2 | 3.8 | 38.9 | 61.1 | 0 |
| Gait disorder | 3.8 | 96.2 | 0 | 0 | 100 | 0 |
| Tremor | 61.5 | 38.5 | 0 | 72.2 | 27.8 | 0 |
| Breathing | 50 | 50 | 0 | 72.2 | 27.8 | 0 |
| Tail Stiffening | 11.5 | 88.5 | 0 | 77.8 | 22.2 | 0 |

### Supplementary Table 2. Time to First Symptom and Time to Culling in *Stx6*^+/+^;h*Tau*^P301S/P301S^ and *Stx6*^-/-^;h*Tau*^P301S/P301S^ Mice. Results of the Kaplan-Meier survival analysis and log rank test assessing differences in time to humane culling or time to first symptom based on *Stx6* expression level. Median time (days) is detailed with corresponding 95% confidence intervals (CI).

|  | **Genotype** | **N start** | **N events** | **Median (days)** | **Lower**  **95% CI** | **Upper 95% CI** | **Difference (days)** | **P-Value Log Rank Test** |
| --- | --- | --- | --- | --- | --- | --- | --- | --- |
| **Time to Cull** | ***Stx6^+/+^*;h*Tau*^P301S/P301S^** | 20 | 20 | 211 | 207 | 225 | -8 | 0.258 |
|  | ***Stx6*^-/-^;h*Tau*^P301S/P301S^** | 20 | 19 | 203 | 198 | 220 |  |  |
| **First Symptom** | ***Stx6^+/+^*;h*Tau*^P301S/P301S^** | 20 | 20 | 172 | *170* | *175* | +5 | 0.154 |
|  | ***Stx6*^-/-^;h*Tau*^P301S/P301S^** | 20 | 20 | 177 | 172 | 178 |  |  |
