# Supplementary material for: Modification of early behavioural, physiological and neuropathological endpoints by syntaxin-6 knockout in a humanised P301S transgenic model of tauopathy
Source: Acta Neuropathol. 2026 Apr 22;151(1):44. doi: 10.1007/s00401-026-03009-2 (PMC13102793; doi:10.1007/s00401-026-03009-2)
Supplement: Supplementary file 2 — Supplementary material (DOCX 62 KB) [file 401_2026_3009_MOESM2_ESM.docx]

# Supplementary Methods

**Statistical Approach**

The broad statistical approach for each study is outlined but the specific ‘n’ number and any deviations in the statistical analyses are detailed for each specific experiment in the relevant figure legend. All graphing and statistical analysis were conducted in Graphpad Prism or InVivoStat.

**Research Governance**

Work with mice was performed under approval and license granted by the UK Home Office (Animals (Scientific Procedures) Act 1986), which conforms to UCL institutional guidelines and Animal Research: Reporting of In Vivo Experiments (ARRIVE) guidelines (www.nc3rs.org.uk/ARRIVE/).

**General Animal Maintenance and Husbandry**

**Housing Conditions and Animal Care**

As described in Hill et al. (2025)^1^, breeding colonies were kept in individually ventilated cages at a temperature of 20-24°C, humidity of 45-65%, an average of 75 air exchanges per hour in the cages and a 12/12-hour light/dark cycle with the lights on at 7AM. The maximum caging density was five mice starting from weaning. Lignocel or EcoPure wood fibres were provided as bedding along with ‘Bed r’ nest’ nesting materials, wood blocks and a shelter.

Mice were fed a standardised irradiated mouse diet and provided with reverse osmosis drinking water *ad libitum.* All materials, including cages, lids, feeders and water bottles were washed in a cage washer and autoclaved before use. All mice were frequently checked by veterinary and animal care staff and had not undergone prior procedures. All animals otherwise received no procedures except those reported in this work. Work was conducted in a CL3 facility, with staff required to air shower upon entry and exit. External animals were only brought in from other clean-screened units and following in-house vet approval.

**Genotyping**

DNA was extracted from ear biopsies of mice using MyTaq Extract-PCR Kit with a 30 min incubation at 75°C followed by a 15 min incubation at 95°C. Genotyping for syntaxin-6 was conducted as described in Hill et al. (2025)^1^. Copy count assays were used for genotyping using the housekeeping gene *Dot1l* as an internal control. In brief, 3 µL DNA was added to 1x TaqPath™ ProAmp™ Master Mix with 1x PrimeTime qPCR assays (1:2 probe:primer ratio) in duplex in a MicroAmp™ Optical 96-Well Reaction Plate (total volume: 20 µL). Reactions were run on the Quant Studio 12K FLEX machine using Quantstudio 12K Flex Software v1.3 (95°C for 10 min, 40x(95°C for 15 sec, 60°C for 1 min). Relative expression of the mutant tau allele was calculated using double delta C_t_ analysis.

**Breeding Strategy**

*Stx6^-/-^* mice on the C57BL/6N background^2^ were crossed with Thy1-hTau.P301S mice^3^ (referred to as h*Tau*^P301S/P301S^ mice hereafter) to generate a fully heterozygote cohort, which were subsequently intercrossed. These offspring were used to establish littermate-derived *Stx6^+/+^;*h*Tau*^P301S/P301S^, *Stx6^-/-^;*h*Tau*^P301S/P301S^, *Stx6^+/+^;*m*Tau*^+^*^/+^* and *Stx6^-/-^;mTau^+/+^* animals to populate the experiment with lines subsequently being maintained as homozygous x homozygous breeding pairs. General animal husbandry and genotyping was conducted as described previously**,** however due to the severity of the phenotype of the h*Tau*^P301S/P301S^ mice, the breeding life span of females was restricted to 3 litters only or if either breeder was observed to have first signs of the disease phenotype.

**Study Design, Power Calculations & Statistical Analysis**

For animals undergoing long-term phenotypic assessment, power calculations were conducted for the primary clinical endpoint, which determined that an n=20 per arm would provide 90% power to detect a 7% change in time to cull. This provided sufficient animals for robust behavioural characterisation whilst accounting for incurrent loss of animals. This was a mixed sex study in line with 3Rs. A single animal was considered an experimental unit. In addition to harvesting animals at clinical endpoint, age-matched time culls were also performed at 1 month, 2 months, 3 months and 5 months (n=10/genotype).

One *Stx6*^-/-;^h*Tau*^P301S/P301S^ animal was culled due to an eye defect at 6.8 months and was censored in survival analysis but data points were included for the behavioural analysis. Two *Stx6*^+/+^;m*Tau*^+/+^ animals were prematurely culled due to unrelated health concerns, resulting in missing rotarod data.

*Statistical Analyses*

The main experimental comparison was pre-defined to be between *Stx6*^+/+^;h*Tau*^P301S/P301S^ and *Stx6*^-/-;^h*Tau*^P301S/P301S^ mice. As secondary comparisons, *Stx6*^+/+^;m*Tau*^+/+^ and *Stx6*^-/-;^m*Tau*^+/+^ mice were compared to inform on baseline deficits, as well as a comparison between *Stx6*^+/+^;m*Tau*^+/+^ and *Stx6*^+/+^;h*Tau*^P301S/P301S^ mice to inform on the diseaserelevance of the readout.

Unless stated otherwise in the figure legend, statistical differences were assessed by one-way ANOVA followed by Fisher’s LSD test of the pre-planned comparisons stated above. To satisfy the assumptions of ANOVA, transformations were conducted in some cases, as detailed in the figure legends. For neuropathological studies assessing multiple brain regions for each animal, a 2-way repeated measures mixed model approach was used for statistical analysis using the unstructured covariance structure to model the within-subject correlations, with genotype as the treatment factor and brain region as the repeated factor. Graphing and statistical analysis were conducted in Graphpad Prism and InVivoStat.

**Rotarod Analysis to Assess Motor Function**

Rotarod performance was assessed monthly (mixed sex) as previously described^4^ with the weight of each animal being recorded prior to each session. For the first month, training sessions were conducted over 3 consecutive days to allow mice to reach a steady baseline level of performance, which was subsequently reduced to 1 day of training prior to the test day in the subsequent months.

The rotarod apparatus was placed in a quiet space with mice being acclimatised to the new environment prior to testing. Each training session consisted of four trials at a constant speed (20 rpm) for a maximum of 60 sec, with the latency to fall being manually recorded within this time period. In the testing session, mice underwent two trials at 5 increasing speed levels (8, 16, 24, 32, 40 rpm) for a maximum of 60 sec. The 24 rpm speed was tested first. Providing all animals passed, they were tested at the higher speeds and assumed to pass the lower speeds. If there was a <100% pass rate, the lower speeds were subsequently assessed. The mean latency to fall (for the two trials at each speed level) was calculated and used in subsequent analysis.

At 6 months, animals with clasping hindlimbs were not assessed on the rotarod for welfare reasons and were imputed as zero for statistical analysis. Two *Stx6^+/+^;*m*Tau^+/+^* mice were culled due to unrelated health problems so were not tested on months 5 and 6. The data points proceeding the deaths of these animals were still included. All genotypes and sexes were tested in the same session, at the same time of the day each month, with the experimenter blind.

**Ink Blot Analysis for Gait Assessment**

Footprint assessment was performed in animals at 5.5 months of age (mixed sex) to detect gait abnormalities. Here, the paws of mice were inked with a nontoxic dye before they were released on one end of an absorbent paper track, which had a width of 10 cm. The base of each pawprint was marked to provide a point of reference for the subsequent measurements taken.

Each gait parameter (n=12) was analysed using one-way ANOVA, with genotype as the treatment factor and sex as a blocking factor. This was followed by the planned comparison between *Stx6*^+/+^;h*Tau*^P301S/P301S^ and *Stx6*^+/+^;m*Tau*^+/+^ mice to prioritise the most robust disease-relevant gait parameters with a deficit (set P-value threshold = 0.0042; unidirectional selection of measurements). These following parameters were used to assess for phenotypic rescue in *Stx6*^-/-^;h*Tau*^P301S/P301S^ mice: Stride Length (RH-RH), Stride Length (LH-LH), Ipsilateral Apart (RF_RH) and Contralateral Difference (Fore). Abbreviations: L/R = left/right; H = hind; F = fore.

To calculate the composite score, the population mean (x̄) and standard deviation (SD) were calculated for each prioritised disease-relevant measurement across all groups. Subsequently, a Z score for each parameter was calculated for each animal, where z= (x-x̄)/SD, where x was the mean of each individual animal. An average Z score for each mouse was then calculated, with data finally being analysed using one-way ANOVA with genotype as the treatment factor and sex as a blocking factor followed by planned comparisons.

**Frailty Assessment**

In a separate cohort of mice, *Stx6*^+/+^;h*Tau*^P301S/P301S^ (n=26), *Stx6^-^*^/-^;h*Tau*^P301S/P301S^ (n=18), *Stx6*^+/+^;m*Tau*^+/+^ (n=5) and *Stx6*^-/-^;m*Tau*^+/+^ (n=5) underwent frailty assessment in three batches (mixed sex)**.** Specifically, mice were assessed for the presence, absence and severity of the following 18 frailty characteristics: alopecia, loss of fur colour, dermatitis, righting reflex, coat condition, piloerection, eye discharge/swelling, microphthalmia, cataracts, nasal discharge, body condition, kyphosis, impaired gait during free walking, tremor, breathing rate/depth, tail stiffening, vestibular disturbance and distended abdomen. This represented a condensed set of parameters derived from a validated, established scoring system described in Rizzo *et al.* (2018)^5^. A 3-point score was assigned to each parameter: no deficit (0), mild deficit (0.5) or severe deficit (1). The assessment was conducted by the same experienced technicians who were blind to genotype. Mice were evaluated for each measure independently followed by calculation of a cumulative score of all measures for each animal (max score = 18).

**Clinical Outcome Measures**

Due to the severe phenotype of the h*Tau*^P301S/P301S^ mice, from 5 months of age they underwent physical health checks twice a week. Upon noticing visible weight loss or abnormal gait (recorded as the time of first symptom), weighing commenced to establish a baseline followed by weighing twice per week. Once an animal lost 15% body weight, weighing was performed daily and their diet was converted to a wet mash diet. Animals were culled and tissue collected as described previously when they reached the 20% weight loss endpoint or if they developed full hind limb paralysis prior to the weight loss endpoint. Animal technicians observing symptom development were blinded to *Stx6* genotype.

Kaplan-Meier survival analysis followed by the Log-Rank test was performed to assess differences in time to first symptom and time to cull between *Stx6*^+/+^;h*Tau*^P301S/P301S^ and *Stx6*^-/-^;h*Tau*^P301S/P301S^ mice. These analyses were conducted with sexes combined given Mann-Whitney testing of sex differences revealed no differences.

**Tissue Collection**

All animals were culled at clinical endpoint or at a 3- or 5-month elective timed cull. Animals were sacrificed by CO_2_ asphyxiation. Brains were removed, dissected on the sagittal plane with the right hemisphere flash frozen and stored at -80^o^C, whilst the left hemisphere was fixed in 10% (v/v) formal buffered saline for a period ranging from 9 to 16 days. Spinal columns were fixed in 10% (v/v) formal buffered saline for a period ranging from 9 to 16 days and decalcified by rolling in 0.5M EDTA solution for a period ranging 7 to 10 days. Following a further 7 to 10 days in fixative, they were dissected into segments and processed into 3 paraffin blocks representing the cervical, thoracic and the lumbar region, with the thoracic region being used for staining. Terminal blood was taken via post-mortem cardiac puncture and serum prepared by centrifuging for 10 min at 2600 rpm after allowing 10 min for clotting in Sarstedt Microvette Serum tubes.

**Neuropathological Assessment**

Brains were processed and stained as described in Hill *et al*. (2025)^1^ with some modifications. The fixed left hemisphere of the brain was embedded in paraffin wax followed by serial sectioning (4 μm nominal thickness) as previously described^6,7^. Sections were then deparaffinised prior to investigation of microgliosis (Iba1 antibody; 1:1000 or 1:250), astrogliosis (GFAP antibody: 1:1000), synapse loss (anti-synaptophysin antibody; 1:5000), neuronal loss (anti-NeuN; 1:3000), phosphorylated tau (AT8-Biotin; 1:50) and misfolded tau (MC1, 1:100) on the Ventana Discovery XT automated IHC staining machine with haematoxylin as the counterstain. Sections were treated using Ventana proprietary detection reagents before staining utilizing 3,3′-diaminobenzidine tetrahydrochloride as the chromogen (DAB Map Detection Kit). The Gemini AS Automated Slide Stainer was used for haematoxylin staining using a conventional approach. Slides were digitally scanned on a NanoZoomer 360 at ×40 magnification, images captured from the NDP.serve3 or NZConnect software and composed with Microsoft Powerpoint.

All brain samples within each experimental group were processed together and stained in a single run, thereby minimising variability. Due to instrument capacity limitations, spinal cord samples were processed in multiple runs with each spine being divided into three blocks. This ensured that all tissue regions were appropriately represented and processed under consistent conditions.

Immunostaining was quantified using QuPath (v0.4.3) software with the experimenter blinded to the genotype of the sections. Following manual annotation of brain regions, colour deconvolution was performed followed by thresholding to select regions positive for DAB staining allowing calculation of the % area stained relative to total tissue area analysed.

NeuN-Positive Cell Density Image Analysis at 3 and 5 Months

NeuN-positive cell density was quantified in the superficial cortex as a previous study demonstrated neuronal loss in this region^8^. The cortex was manually annotated using NZConnect, with colour deconvolution subsequently being performed using QuPath. Then a machine learning-based pixel classifier was trained to distinguish tissue sections from glass and create whole tissue annotations. The region of interest (ROI) that was 500 μm deep from the cortical surface was obtained by first getting the ROI difference between the whole tissue annotation and the tissue annotation eroded by 500 μm. This band around the edge of the tissue section was then intersected with the cortex annotations to limit the ROI to within the cortex. Within the resulting annotation, Cellpose^9^ was used to detect DAB-positive cells from the DAB deconvolved channel. Finally, an object classifier was applied to the resulting detections from Cellpose to filter out non-neuronal staining.

NeuN-positive cell density was calculated by:

$$\mathrm{NeuN}^{+}Cells/\mathrm{mm}^{2}=\frac{NeuN Cell Counts}{Area of Superficial Cortex}x {10}^{6}$$

**Biochemical Analysis**

*Total Brain Homogenate Preparation*

As described in Hill *et al.* (2025)^1^, 20% (w/v) homogenates were prepared in DPBS by ribolysing with 1.4 mm ceramic homogenisation beads at 6,500 rpm for 45 sec using the Ribolyser Precellys 24. Following a 30 min incubation on ice, homogenates were distributed into a 20% (w/v) stock and a 10% (w/v) stock with 1x final Halt™ Protease and Phosphatase Inhibitor Cocktail. Aliquots of brain homogenates were prepared in prelubricated tubes to avoid multiple freeze-thaw cycles. Brain homogenate total protein was quantified using Pierce™ BCA Protein Assay kit according to the manufacturer’s instructions.

*Immunoblotting*

10% (w/v) homogenates with protease/phosphatase inhibitors (mixed sex) were clarified by centrifuging at 1,000 *g* for 5 min at 4°C with the supernatant being used for sample preparation.

For immunoblotting, lysates were diluted in DPBS and 4x Laemelli sample buffer to obtain a final 1x concentration with 355 mM final 2-mercaptoethanol. Following boiling at 95°C for 5 min, 30 μg total protein was loaded onto a 4-12% Bis-Tris polyacrylamide gel in addition to the SeeBlue Protein ladder. Following electrophoresis at 150V for 3 hr, protein was electroblotted onto a nitrocellulose membrane at 35 V for 2 hr. Odyssey Blocking Buffer (TBS) was used for blocking and antibody dilution steps and TBS with 0.1% tween (TBST) being used for washing steps. Phospho-tau species were detected first (AT8 or PHF1 at 1:500) following further immunodetection of total tau (K9JA, 1:5000) and β-actin (A5441, 1:5000). For secondaries, IRDye 800CW Donkey anti-rabbit IgG was used at a 1:5000 dilution and the IRDye 680RD goat anti-mouse IgG antibody at a 1:20,000 dilution.

Image Studio™ Software was used to quantify the fluorescent signals from the 700 nm and 800 nm channels. Rectangles were drawn around the target bands as well as the loading control bands. Median background subtraction method was used. Following quantification, the *Lane Normalisation Factor* (LNF) was calculated as follows:

$$LNF=\frac{\beta-Actin Signal for Each Lane}{\beta-Actin Signal from Lane with Highest \beta-Actin Signal}$$

The normalised signal for total tau (K9JA) was then calculated using the following formula:

$$Normalised Signal=\frac{Target Signal for Each Lane}{LNF for Each Lane}$$

This was subsequently used to correct the signal for the AT8/PHF1 tau signal. Finally, all values were normalised to an average of the *Stx6^+/+^;*h*Tau*^P301S/P301S^ animals.

**Assessment of Seeding Differences Using Tau RD P301S FRET Biosensor Cells**

Tau RD P301S FRET biosensor cells were housed in an incubator at 37°C/5% CO_2._ Cells were cultured in DMEM + GlutaMAX-I + 10% Fetal Bovine Serum (FBS) + 1% penicillin-streptomycin (PS).

Brain homogenates were prepared as described previously as 10% (w/v) brain homogenates with protease and phosphatase inhibitors. These were subsequently clarified at 20,000 g for 15 min with the supernatant being transferred to single-use aliquots and total protein being quantified by BCA.

One day prior to treatment, Tau RD P301S FRET biosensor cells were seeded at 25,000 cells/well in 130 µL media into TPP 96-well plates. The following day, a dilution series of clarified 10% (w/v) brain homogenates were diluted in reduced-serum Opti-MEM*.* Diluted samples were subsequently mixed with an equal volume Opti-MEM supplemented with lipofectamine 2000 (1:8 dilution). Following a 20-30 min incubation at RT, 20 µL of the transfection mix was added to the cells at ~70% confluency, which were subsequently incubated at 37°C and 5% CO_2_ within an IncuCyte S3 live cell imaging apparatus.

IncuCyte S3 Software (2022B/Rev2) acquired images using the 20× objective lens every 3 hr (16 images/well, same well coordinates) in phase contrast and green channels. Image analysis was performed using the Basic Analyzer module, employing a phase mask to quantify cell confluency and the green mask to quantify bright, punctate aggregates (FRET detected in GFP channel due to spectral overlap) as opposed to the baseline diffuse green background. Typical parameters for the green channel were as follows: segmentation - top-hat; radius- 10 µm; threshold- 4.75 GCU; edge split off.

**Biomarker Assessment**

Serum NfL levels were measured at 3 months, 4 months and 5 months in singulate using the single-molecule-array (Simoa) NF-light V2 Advantage Kit according to the manufacturer’s instructions. This was run on the Simoa HD-X platform with the experimenter’s blind to study design.

**Key Resources Table**

| REAGENT or RESOURCE | SOURCE | IDENTIFIER |
| --- | --- | --- |
| Antibodies | | |
| Anti-β-Actin Antibody [AC-15] | Sigma | A5441; RRID:AB_476744 |
| Anti-Iba1 Antibody | AlphaLaboratories | Cat#019-19741; RRID:AB_839504 |
| Anti-GFAP Antibody | Dako, Agilent | Z0334; RRID:AB_10013382 |
| Anti-Synaptophysin Antibody | Invitrogen | PA5-27286; RRID:AB_2544762 |
| AT8 Biotin Antibody | Invitrogen | MN1020B; RRID:AB_223648 |
| Anti-NeuN Antibody | Abcam | Ab177487; RRID:AB_2532109 |
| Anti-Tau Antibody [K9JA] | DAKO | A0024; RRID:AB_10013724 |
| Anti-Tau Antibody [MC1] | Peter Davies (Described in Jicha *et al.* (1997)^10^) | NA |
| Anti-pTau Antibody [PHF1] | Peter Davis (Described in Greenberg *et al.* 1992)^11^) | NA |
| Anti-pTau Antibody [AT8] | Invitrogen | MN1020; RRID:AB_223647 |
| Biotinylated Polyclonal Rabbit Anti-Mouse Immunoglobulin Secondary Antibody | Dako; Agilent | 315-065-045-JIR |
| Goat Anti-rabbit secondary antibody | Abcam | Ab6720  RRID: AB_954902 |
| IRDye 800CW donkey anti-rabbit | LI-COR | 926-32213; RRID:AB_621848 |
| IRDye 680RD goat anti-mouse IgG | LI-COR | 926-68070; RRID:AB_10956588 |
| Chemicals, peptides, and recombinant proteins | | |
| Lipofectamine™ 2000 Transfection Reagent | Thermo Fisher | 11668030 |
| Halt Protease & Phosphatase Inhibitor Cocktail (100X) | Thermo Scientific | 78444 |
| DAB Map Detection Kit | Roche Tissue Diagnostics | 05266360001, 760-124 |
| Critical commercial assays | | |
| Pierce BCA Protein Assay Kit | Thermo Scientific | 23227 |
| NF-light V2 Advantage Kit | Quanterix | 104073 |
| Experimental models: Cell lines | | |
| Human: Tau RD P301S FRET Biosensor Cell Line | ATCC | CRL-3275 |
| Experimental models: Organisms/strains | | |
| Mouse: C57BL/6NTac-Stx6^em1(IMPC)H^ | UKRI-MRC Harwell as part of the IMPC () | Allele MGI ID: 6266827; EM:12503 |
| Mouse: Thy1-hTau.P301S (CBA.C57BL/6J) | Laboratory of Michel Goedert (Described in Allen *et al.* (2002)^3^ | NA |
| Oligonucleotides | | |
| Primers for PCR Genotyping of *Stx6^+/+^*, *Stx6^+/-^* and *Stx6^-/-^* mice: CGATCTGTGAGACTCATCGGG, GGGAGTCCTAACACCACCTTC, GGACACCATGCTTTCAAGATTT. | Jones *et al.* (2024)^2^ | NA |
| Dot1l PrimeTime qPCR Assay: Forward Primer, GTT GGC ATC CTT ATG CTT CAT C; Reverse Primer, GTG TGC TAC GCC TGA AAT AAA G; Probe, /5SUN/TT GAG AGC T/ZEN/G GCC CTG AAT GGT C/3IABkFQ/ | IDT | NA |
| P301S PrimeTime qPCR Assay: Forward Primer, TCCTAGAATTGATCCTGGCGTAA; Reverse Primer, AACTGGGAGTCGCTACAGTGAGT; Probe, FAM-AGCGAAGAGGCCCGCACCTCA-BHQ | Laboratory of Michel Goedert | NA |
| Software and algorithms | | |
| ImageLab 6.0 | Biorad | https://www.bio-rad.com/en-uk/product/image-lab-software |
| NDP.serve3 | NanoZoomer Digital Pathology | https://nanozoomer.unisa.edu.au/ndp/serve/home |
| NZConnect 1.0.36 (IVD) | Hamamatsu | https://nzconnect.ion.ucl.ac.uk/nz/connect/home |
| Qupath (Version 0.4.3) | Bankhead et al.^12^ | https://qupath.github.io/) |
| i-control (Version 3.4.2.0) | Tecan | NA |
| Odyssey Acquisition Software (Version 2.1) | LI-COR | https://www.licor.com/bio/las/ |
| Image Studio Lite Ver 5.2 | LI-COR | Discontinued |
| InVivoStat v4.7.0 | Clark et al. (2012)^13^ | https://invivostat.co.uk/download/ |
| GraphPad Prism 9 | GraphPad | https://www.graphpad.com/features |
| Incucyte 2022B Rev2 | Satorius | https://downloads.essenbioscience.com/get/incucyte-2022b-rev2-gui |

**References**

1 Hill, E. *et al.* Intracellular trafficking SNARE protein, syntaxin-6, modifies prion cellular phenotypes and risk of disease development in vivo. *Acta neuropathologica* **150**, 48, doi:10.1007/s00401-025-02946-8 (2025).

2 Jones, E. *et al.* Characterisation and prion transmission study in mice with genetic reduction of sporadic Creutzfeldt-Jakob disease risk gene Stx6. *Neurobiology of disease* **190**, 106363, doi:<https://doi.org/10.1016/j.nbd.2023.106363> (2024).

3 Allen, B. *et al.* Abundant tau filaments and nonapoptotic neurodegeneration in transgenic mice expressing human P301S tau protein. *The Journal of neuroscience : the official journal of the Society for Neuroscience* **22**, 9340-9351, doi:10.1523/JNEUROSCI.22-21-09340.2002 (2002).

4 Scattoni, M. L. *et al.* Early behavioural markers of disease in P301S tau transgenic mice. *Behav Brain Res* **208**, 250-257, doi:10.1016/j.bbr.2009.12.002 (2010).

5 Sukoff Rizzo, S. J. *et al.* Assessing Healthspan and Lifespan Measures in Aging Mice: Optimization of Testing Protocols, Replicability, and Rater Reliability. *Current Protocols in Mouse Biology* **8**, e45, doi:<https://doi.org/10.1002/cpmo.45> (2018).

6 Jones, E. *et al.* Knockout of Sporadic Creutzfeldt-Jakob Disease Risk Gene in Mice Extends Prion Disease Incubation Time. *bioRxiv*, 2023.2001.2010.523281, doi:10.1101/2023.01.10.523281 (2023).

7 Sandberg, M. K. *et al.* Prion neuropathology follows the accumulation of alternate prion protein isoforms after infective titre has peaked. *Nature Communications* **5**, 4347, doi:10.1038/ncomms5347 (2014).

8 Hampton, D. W. *et al.* Cell-mediated neuroprotection in a mouse model of human tauopathy. *The Journal of neuroscience : the official journal of the Society for Neuroscience* **30**, 9973-9983, doi:10.1523/jneurosci.0834-10.2010 (2010).

9 Stringer, C., Wang, T., Michaelos, M. & Pachitariu, M. Cellpose: a generalist algorithm for cellular segmentation. *Nature methods* **18**, 100-106, doi:10.1038/s41592-020-01018-x (2021).

10 Jicha, G. A., Bowser, R., Kazam, I. G. & Davies, P. Alz-50 and MC-1, a new monoclonal antibody raised to paired helical filaments, recognize conformational epitopes on recombinant tau. *Journal of neuroscience research* **48**, 128-132, doi:10.1002/(sici)1097-4547(19970415)48:2<128::aid-jnr5>3.0.co;2-e (1997).

11 Greenberg, S. G., Davies, P., Schein, J. D. & Binder, L. I. Hydrofluoric acid-treated tau PHF proteins display the same biochemical properties as normal tau. *The Journal of biological chemistry* **267**, 564-569 (1992).

12 Bankhead, P. *et al.* QuPath: Open source software for digital pathology image analysis. *Scientific Reports* **7**, 16878, doi:10.1038/s41598-017-17204-5 (2017).

13 Clark, R. A., Shoaib, M., Hewitt, K. N., Stanford, S. C. & Bate, S. T. A comparison of InVivoStat with other statistical software packages for analysis of data generated from animal experiments. *J Psychopharmacol* **26**, 1136-1142, doi:10.1177/0269881111420313 (2012).
